# Involvement of Hypothalamic AMP-Activated Protein Kinase in Leptin-Induced Sympathetic Nerve Activation

**DOI:** 10.1371/journal.pone.0056660

**Published:** 2013-02-13

**Authors:** Mamoru Tanida, Naoki Yamamoto, Toshishige Shibamoto, Kamal Rahmouni

**Affiliations:** 1 Department of Physiology II, Kanazawa Medical University, Uchinada, Ishikawa, Japan; 2 Department of Biomedical Sciences, College of Life Sciences, Ritsumeikan University, Kusatsu, Shiga, Japan; 3 College of Pharmacology, Ritsumeikan University, Kusatsu, Shiga, Japan; 4 Departments of Pharmacology and Internal Medicine, University of Iowa Carver, College of Medicine, Iowa City, Iowa, United States of America; Max Delbrueck Center for Molecular Medicine, Germany

## Abstract

In mammals, leptin released from the white adipose tissue acts on the central nervous system to control feeding behavior, cardiovascular function, and energy metabolism. Central leptin activates sympathetic nerves that innervate the kidney, adipose tissue, and some abdominal organs in rats. AMP-activated protein kinase (AMPK) is essential in the intracellular signaling pathway involving the activation of leptin receptors (ObRb). We investigated the potential of AMPKα2 in the sympathetic effects of leptin using in vivo siRNA injection to knockdown AMPKα2 in rats, to produce reduced hypothalamic AMPKα2 expression. Leptin effects on body weight, food intake, and blood FFA levels were eliminated in AMPKα2 siRNA-treated rats. Leptin-evoked enhancements of the sympathetic nerve outflows to the kidney, brown and white adipose tissues were attenuated in AMPKα2 siRNA-treated rats. To check whether AMPKα2 was specific to sympathetic changes induced by leptin, we examined the effects of injecting MT-II, a melanocortin-3 and -4 receptor agonist, on the sympathetic nerve outflows to the kidney and adipose tissue. MT-II-induced sympatho-excitation in the kidney was unchanged in AMPKα2 siRNA-treated rats. However, responses of neural activities involving adipose tissue to MT-II were attenuated in AMPKα2 siRNA-treated rats. These results suggest that hypothalamic AMPKα2 is involved not only in appetite and body weight regulation but also in the regulation of sympathetic nerve discharges to the kidney and adipose tissue. Thus, AMPK might function not only as an energy sensor, but as a key molecule in the cardiovascular, thermogenic, and lipolytic effects of leptin through the sympathetic nervous system.

## Introduction

Leptin, a hormone produced in white adipose tissue (WAT), is released into the blood and acts on the central nervous system to reduce appetite and increase energy expenditure [1], [2]. Consistent with this function, leptin increases sympathetic nerve activity (SNA) to thermogenic brown adipose tissue [2] and the kidney, leading to arterial pressure elevation [3], [4], [5]. In addition, leptin has been implicated as a key factor linking obesity and hypertension through sympatho-activation [4].

Numerous intracellular signaling pathways are involved in the cardiovascular and metabolic effects of leptin in the hypothalamus [3], [6]. AMP-activated protein kinase (AMPK), comprising 3 subunits: α, β and γ [6], is an important component in glucose and lipid metabolism and is dependent on the intracellular AMP/ATP ratio. Thus, AMPK can act as an efficient sensor of the cellular energy state in regulating feeding behavior [6], [7]. Interestingly, leptin appears to modulate the activity of AMPKα2, but not of AMPKα1, thereby resulting in a reduction in food intake and body weight [7]. In addition, mice lacking AMPKα2 in AgRP or POMC neurons exhibit contrasting metabolic phenotypes, highlighting the importance of hypothalamic AMPKα2 for energy homeostasis [8].

Recent investigations demonstrated that hypothalamic AMPK plays crucial roles not only in regulating energy homeostasis but also in glucose metabolism [9], sleep [10] and cardiovascular control [11]. For example, central injection of 5-aminoimidazole-4-carboxamide 1-beta-D-ribofuranoside (AICAR), an AMPK activator, or compound C, an AMPK inhibitor, alters glucose production in the liver [9] and neural activity in sleep-related areas of the brain [10]. Moreover, mice lacking AMPKα2 exhibit elevated arterial pressure [11]. In addition, injection of triiodothyronine into the hypothalamus decreases AMPK activity and stimulates sympathetic nerve activity that innervates BAT [12]. We found that intracerebroventricular (ICV) injection of compound C stimulated the sympathetic nerve outflow to the kidney, adrenal glands and BAT in a dose-dependent manner [13], similar to the sympathetic nerve outflows observed after ICV injection of leptin [2], [3], [4], [5]. Therefore, AMPK may be a hypothalamic intracellular signaling factor and a mediator of SNA responses induced by leptin action in the hypothalamus.

In the present study, to investigate the possible role of AMPKα2 in the hypothalamus in the control of the autonomic nervous system by leptin, we generated an AMPKα2 knockdown rat model using an *in vivo* siRNA silencing approach. We examined the effects of ICV injection of leptin on regional SNA in control and AMPKα2 siRNA-treated rats. We also examined the consequence of silencing hypothalamic AMPKα2 on leptin effects on feeding behavior, lipolysis markers in the peripheral blood and cardiovascular regulation.

## Methods

### Animals

Male Wistar rats, weighing 250–270 g, were housed in a room maintained at 24±1°C and illuminated for 12 h (08:00–20:00) everyday. Food and water were freely available. Rats were allowed to adapt to the environment for at least 1 week before the experiment. All animal care and handling procedures were approved by the Institutional Animal Care and Use Committee of Ritsumeikan University.

### Transfection of siRNA in the Rat Brain

To knockdown AMPKα2 in the hypothelamus, we performed in vivo siRNA transfection in Wistar rats according to a previous study [14]. Under anesthesia, induced by intraperitoneal (IP) injection of ketamine (91 mg/kg) and xylazine (9.1 mg/kg), a 23-gauge stainless steel guide cannula with a stylet was implanted into the third cerebral ventricular area using a stereotaxic apparatus (coordinates: AP, 1.0 mm posterior to the bregma; L, 0 mm; V, 7.5 mm) as reported previously. After 7–10 days recovery, the stylet was removed from the guide cannula, and a 29-gauge injection cannula attached to a 25 µl Hamilton syringe was inserted. The injection cannula was designed to be 1.0 mm longer than the guide cannula in order to reach the floor of the third ventricle. According to the manufacture's instructions, 1 µL of 10 µg/µL AMPKα2-siRNA (Invitrogen Corporation,10620312) or control-siRNA (invitrogen Corporation,10620312-Negacon) duplex stock solution was incubated with 10 µL Invivofectamine Reagent (Invitrogen Corporation, 1377-901) for 30 minutes at room temperature in an orbital shaker. Then the invivofectamine–siRNA mixture was diluted with 15 volumes (150 µL) of 5% glucose. Five microliters of the diluted mixture was delivered into the third cerebral ventricle. After injection, rats were returned to their cages and given ad libitum access to food and water. ICV injection was repeated during 3 days (2 time per day).

### Western Blotting and Determination of Blood Levels of Catecholamine, Glucose, Leptin

To examine the hypothalamic knockdown of AMPKα2, we determined protein expression levels of AMPKα2 by western blotting. Next day after 3days treatment of siRNA, rats were killed by decapitation and hypothalami quickly removed and homogenized on ice, and blood was sampled for determination of plasma levels of adrenaline, noradrenaline, glucose and leptin. The amount of total protein was measured for each sample by the bicinchoninic acid method. 10 µg of total proteins per sample were loaded and separated by SDS-PAGE (8% acrylamide) before transfer onto polyvinylidene difluoride membranes. Membranes were then sequentially blocked in blocking buffer, probed overnight at 4°C with gentle shaking with primary antibody against AMPKα2 (abcam, 1∶1000 dilution) and incubated for 1 h at room temperature with horseradish peroxidase (HRP)-conjugated secondary antibodies against the molecular weight standard (Biotinylated Protein Ladder, Cell Signaling Technology, Inc.) and an HRP labeled anti-rabbit IgG (H+L) antibody (Epitomics, Inc., Burlingame, CA, USA). Specific proteins were detected by chemiluminescence with the Phototope Detection system. Western blotting was quantified by densitometry relative to total AMPKα2 using NIH Image software (Image J).

To examine the plasma levels of adrenaline, noradrenaline, glucose and leptin in rats treated ICV AMPKα2-siRNA or control-siRNA, blood samples were transferred immediately to chilled tubes containing EDTA, and then centrifuged (3000 rpm, 12 min, 4°C). The plasma samples were separated and stored at −80°C. Plasma concentrations of adrenaline and noradrenaline were determined by high-performance liquid chromatography with a trihydroxyindole reaction. Plasma leptin levels were determined using a rat leptin RIA kit (Rat Leptin Assay Kit, IBL), and plasma glucose levels were measured with a Fuji Dri-chem system (Fuji Film, Japan).

### Blood FFA Level

Plasma FFA was examined in awake rats as described previously [15]. For this, the 3–4 days before treatment with siRNA, a silicone catheter was implanted under anaesthesia by IP injection of ketamine (91 mg/kg) and xylazine (9.1 mg/kg) in the right external jugular vein, with its end at a point just outside the atrium. Next day after 3days treatment of siRNA, they received ICV injection of leptin. Blood samples were collected at 30-min intervals up to 2 hours after ICV injection and were also collected in 4 hours after injection. The plasma was separated and prepared immediately for the assay of FFA by enzymatic analysis using commercially available kit. FFA was determined by the acyl CoA synthetase-acyl CoA oxidase method using a non-esterified fatty acid C-test Kit (Wako Pure Chemical Industries, Ltd. Cat.# 279-75401).

### Recording of SNA

On the day of the experiment (after 3 days of AMPKα2-siRNA or control-siRNA traetment), food was removed 5 h prior to surgery. Under anesthesia, induced by IP injection of 1.2 g/kg urethane, a polyethylene catheter was inserted into the left femoral vein for intravenous injection. Another catheter was inserted into the left femoral artery for arterial pressure recording. The rat was then cannulated through the trachea and fixed in a stereotaxic apparatus. Body temperature was maintained at 37.0–37.5°C using a heating pad and monitored with a thermometer inserted into the rectum. SNA measurements were done as in our previous studies [13], [16]. To record renal SNA (RSNA), the left renal nerve was exposed retroperitoneally through a left flank incision using a dissecting microscope. To measure brown adipose tissue (BAT-SNA), the left sympathetic nerve innervating interscapular BAT was exposed through a left dorsal incision. For white adipose tissue (WAT-SNA) recording, an abdominal blood vessel supplying the testis and adipose tissue of the epididymis was located and the nerve bundles were exposed.

The proximal side of each nerve was attached on one side of a pair of stainless wire electrodes. The distal end of the respective nerve was ligated, cut and then hooked up to electrodes to record efferent nerve activity (approximate distance between electrode attachment site and ligation site is 1–2 mm). The recording electrodes were fixed with a silicon gel (liquid A & liquid B, Kagawa kikai Co. JAPAN) to prevent dehydration and for electrical insulation. Respective nerves were recorded in separate rats. After surgery, each rat was allowed to stabilize for 30–60 min.

Electrical changes in all nerves were amplified 2000–5000 times with a band path of 100 to 1000 kHz, and monitored by an oscilloscope. Raw data of the nerve activity was converted to standard pulses by a window discriminator, which separated discharge from electrical background noise which was determined post mortem. Both the discharge rates and the neurogram were sampled with a Power-Lab analog-to-digital converter for recording and data analysis on a computer. Background noise, which was determined 30–60 min after the animal was euthanized, was subtracted. Nerve activity was rectified and integrated, and baseline nerve activity was normalized to 100%. Baseline measurements of RSNA, BAT-SNA and WAT-SNA were made 5 min prior to ICV injection of vehicle (PBS solution, 10 µl), leptin (10 µg/10 µl PBS), or MT-II (600 pmol/10 µl PBS). These doses were determined by referring to previous studies [13], [17]. These parameters were recorded for 240 min. At the end of the experiment, hexamethonium chloride (10 mg/kg) was administered intravenously to ensure that post-ganglionic efferent sympathetic nerve activity had been recorded.

### Hemodynamic Measurements

To directly measure arterial pressure in conscious state, each rat was anesthetised with IP injection of mixture of ketamine and xylazine and the catheter in the left femoral artery was connected to a blood pressure transducer (DX-100, Nihon Kohden, Japan), and the output signal of the transducer was amplified in a blood pressure amplifier (AP641G, Nihon Kohden, Japan). Arterial pressure was monitored with an oscilloscope, sampled with the Power-Lab, and stored on a hard disk for off-line analysis to calculate mean arterial pressure (MAP) and heart rate (HR). Upon completion of the surgical procedure, rats were allowed to recover for 4–5 days as described by previous study [18]. Rats received either leptin or vehicle ICV, and measurement of MAP and HR performed for 4 hour after injection.

### Food Intake and Body Weight Study

Body weight and food intake of individually caged control and AMPKα2-siRNA rats were measured before and after treatment. After 3 days of siRNA treatment, rats (n = 8–10 animals per group) were assigned to receive one ICV injection of vehicle (PBS solution, 10 µl) or murine leptin (10 µg/10 µl in PBS) at 6 PM. Body weight and food intake were measured 24 h after ICV leptin or vehicle.

### Statistical Analysis

The RSNA, BAT-SNA and WAT-SNA data measured during each 5 min period after injection of all agents were analyzed by digital signal processing and appropriate statistical analyses. Percent changes from the baseline values were calculated for SNA. All data were expressed as means ± SEM. Student's t test was used to compare basal levels between 2 groups. Analysis of variance (ANOVA) for repeated measurements was used to compare between multiple groups followed by Tukey's post hoc test. A value of *P*<0.05 was considered to be statistically significant.

## Results

### Knockdown of AMPKα2 by siRNA

**Figure 1:**
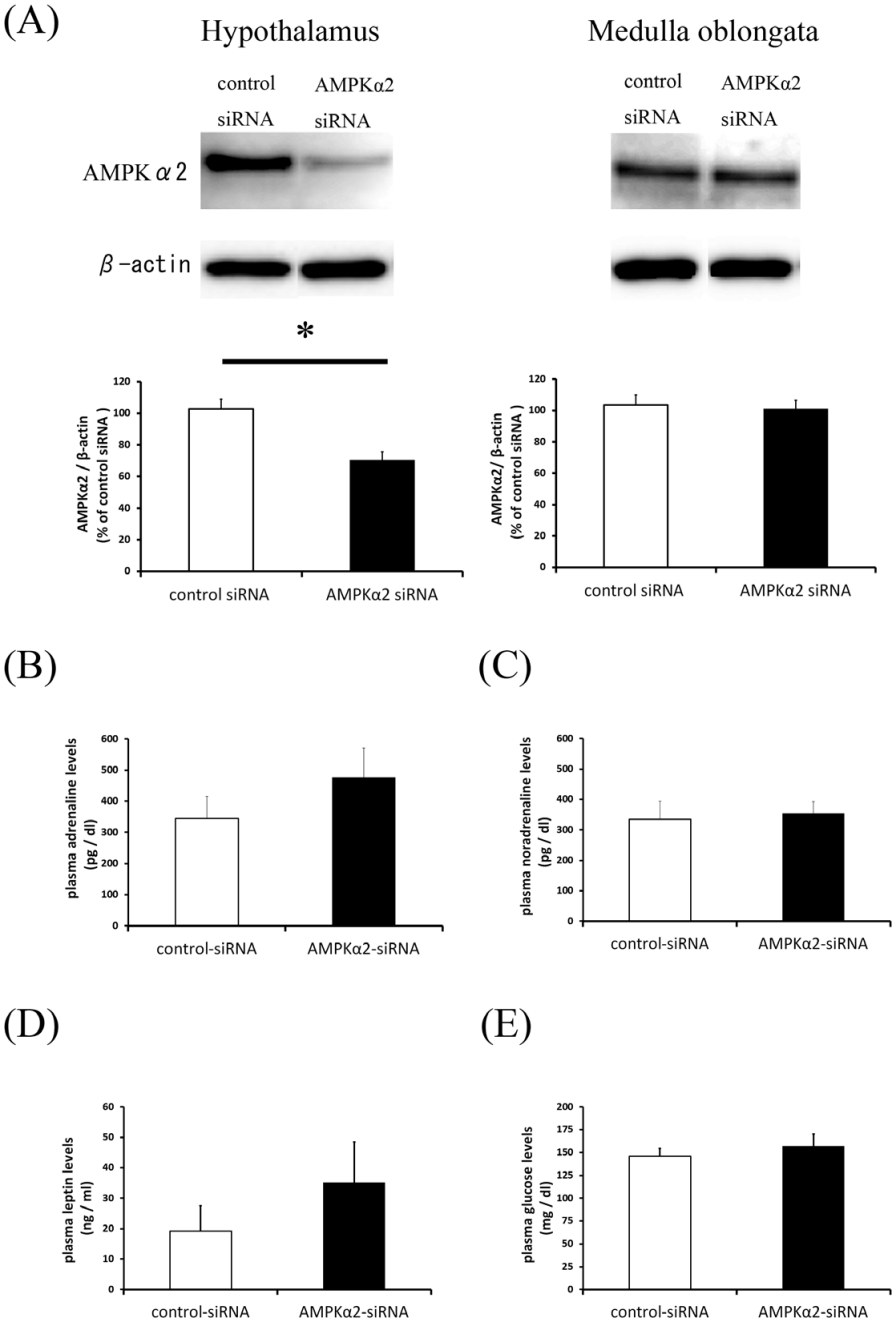
Identification of hypothalamus-specific knockdown of AMPKα2 with siRNA in rats. The protein expression of AMPKα2 in the hypothalamus and medulla oblongata were analyzed by western blotting (A). AMPKα2 siRNA significantly reduced the protein expression level of AMPKα2 in the hypothalamus, but not the medulla oblongata. Comparison of blood adrenaline level (B), blood noradrenaline level (C), plasma leptin level (D) and plasma glucose level (E) between control-siRNA rats and AMPKα2-siiRNA rats. Data present means ± SEM. n = 6 rats per group. *P<0.05 vs. control-siRNA group.

To test the possibility of using siRNA for hypothalamus-specific knockdown of AMPKα2 in rats, AMPKα2 protein levels in the brain were determined by Western blot 3 days after ICV administration of control or AMPKα2 siRNA (Figure 1A). AMPKα2 protein expression in the hypothalamus was decreased in rats treated with AMPKα2 siRNA compared with the control siRNA-treated rats, while AMPKα2 protein expression in the medulla oblongata was not significantly different between the two groups.

Next, we compared the blood levels of adrenaline (B), noradrenaline (C), leptin (D), and glucose (E) in control and AMPKα2 siRNA-treated rats. All parameters were similar between the two groups and significant difference was not detected.

### Hypothalamic AMPKα2 knockdown attenuates the feeding, body weight, lipolysis, and BP responses induced by leptin

**Figure 2:**
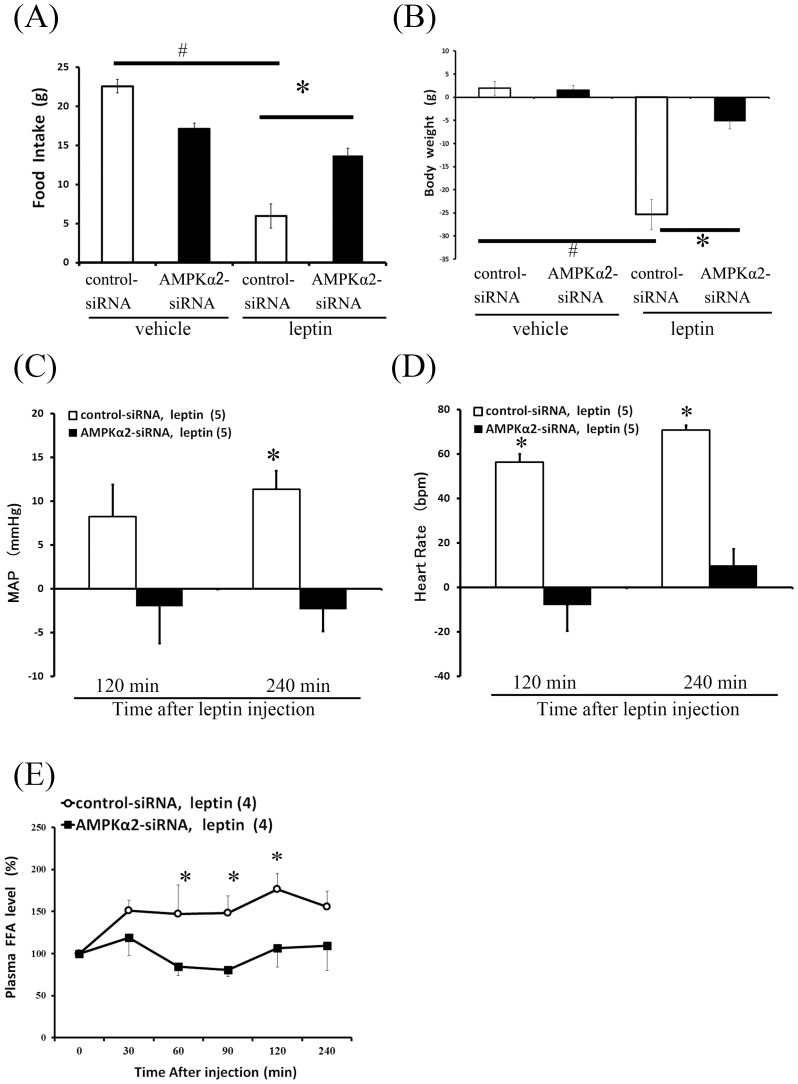
Effect of hypothalamic AMPKα2 siRNA knockdown on feeding, body weight, blood FFA and cardiovascular responses induced by ICV injection of leptin. Effects of ICV injection of leptin on food intake (A) and body weight (B) after hypothalamus-specific knockdown by siRNA of AMPKα2 (black bars) vs. controls (siRNA-control, white bars). Data present means ± SEM. n = 6 rats per group. #P<0.05 vs. vehicle group, *P<0.05 vs. control-siRNA group. Time-course of MAP (C), HR (D) and blood FFA (E) responses to ICV leptin in control-siRNA and AMPKα2-siRNA rats. Data was expressed as means ± SEM. SNA is expressed as a percentage of baseline values (at time 0). Number of animals used is shown in parentheses. *P<0.05 vs. control-siRNA group.

We compared the effects of ICV leptin on appetite, body weight, blood FFA levels, and the cardiovascular function in control and AMPKα2 siRNA-treated rats. As shown in Figure 2A and 2B, in control siRNA-treated rats, ICV injection of leptin caused a significant decrease in body weight and food intake. However, in AMPKα2 siRNA-treated rats, ICV leptin did not significantly affect body weight or food intake, as compared with vehicle. In should be noted that these rats had different basal body weights at 3 days after transfection of control or AMPKα2 siRNA. Prior to injecting leptin or vehicle, AMPKα2 siRNA-treated rats had lower post-transfection body weights (pre-transfection: 348.2 g±5.5 g; post-transfection: 302.5 g±5.7 g), whereas body weights were unchanged in control siRNA-treated rats (pre-transfection: 333.6 g±9.2 g; post-transfection: 334.5 g±8.1 g).

Next, we compared the effects of ICV leptin on cardiovascular function between both groups. In control siRNA-treated rats, leptin induced a significant elevation in MAP and HR as compared with AMPKα2 siRNA-treated rats (Figure 2C and D). Basal levels of MAP (109.2±7.7 mmHg) and HR (426±23 bpm) of AMPKα2 siRNA-treated rats were significantly higher compared with MAP (91.5±4.4 mmHg) and HR (336±18 bpm) of control siRNA-treated rats.

As shown in Figure 2E, in conscious control siRNA-treated rats, ICV injection of leptin caused a significant elevation in the plasma FFA levels at 60–120 min as compared with AMPKα2 siRNA-treated rats. With regard to basal levels prior to injecting leptin, there was no significant difference between these groups in the FFA levels (control siRNA: 0.278±0.033 mEq/L; AMPKα2 siRNA: 0.290±0.05 mEq/L).

### Attenuated sympathetic nerve activity responses to leptin in AMPKα2 siRNA-treated rats

**Figure 3:**
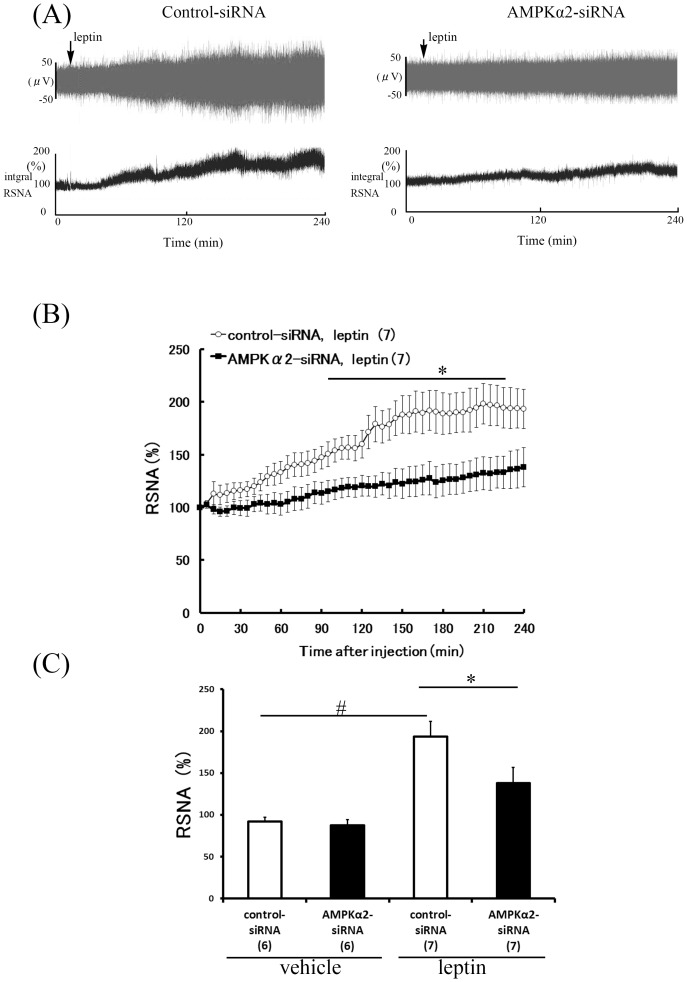
Effect of hypothalamic AMPKα2 siRNA knockdown on RSNA response to ICV leptin. Representative neurogram (A) and time-course (B) of RSNA response to ICV in control-siRNA and AMPKα2-siRNA rats. Bar graph data (C) averaging last hour of recording after leptin or vehicle treatment in both groups are presented as mean ± SEM. of percentages of values at 0 min. Number of animals used is shown in parentheses. #P<0.05 vs. vehicle group, *P<0.05 vs. control-siRNA group.

**Figure 4:**
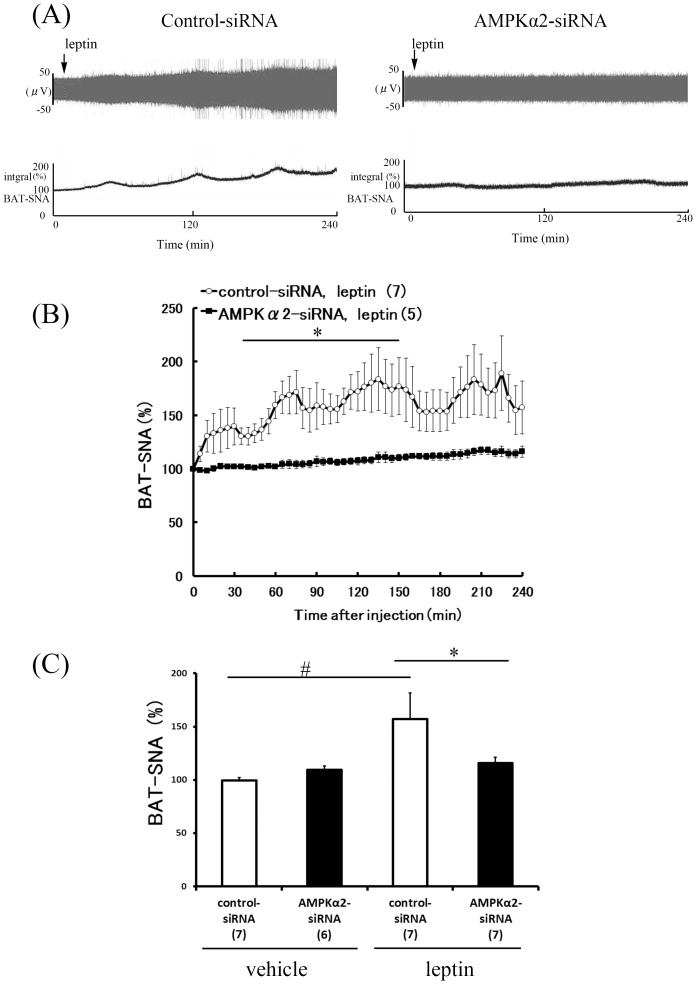
Effect of hypothalamic AMPKα2 siRNA knockdown on BAT-SNA response to ICV injection of leptin. Representative neurogram (A) and time-course (B) of BAT-SNA response to ICV in control-siRNA and AMPKα2-siRNA rats. Bar graph data (C) averaging last hour of recording after leptin or vehicle treatment in both groups are presented as mean ± SEM. Numbers of animals used are shown in parentheses. #P<0.05 vs. vehicle group, *P<0.05 vs. control-siRNA group.

**Figure 5:**
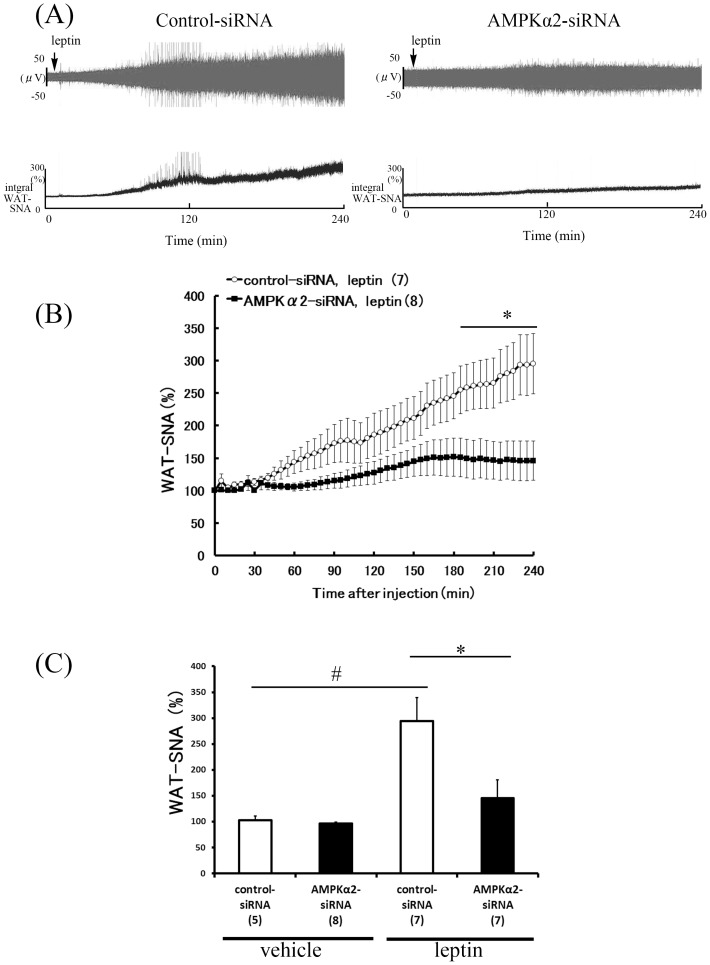
Effect of hypothalamic AMPKα2 siRNA knockdown on WAT-SNA response to ICV injection of leptin. Representative neurogram (A) and time-course (B) of WAT-SNA response to ICV in control-siRNA and AMPKα2-siRNA rats. Bar graph data (C) averaging last hour of recording after leptin or vehicle treatment in both groups are presented as mean ± SEM. SNA. Number of animals used is shown in parentheses. #P<0.05 vs. vehicle group, *P<0.05 vs. control-siRNA group.

To determine a possible role for hypothalamic AMPKα2 in the control of sympathetic nervous system activities by leptin, we compared the effects of ICV injection of leptin on RSNA, BAT-SNA and WAT-SNA between control siRNA-treated and AMPKα2 siRNA-treated rats. In control siRNA-treated rats, ICV leptin (10 µg) caused a significant increase in SNA subserving the kidney (193.4±18.5%, Figure 3), BAT (157.3±24.5%, Figure 4), and WAT (294.4%±45.4%, Figure 5). In AMPKα2 siRNA-treated rats, leptin also increased RSNA (138.1±18.4%), BAT-SNA (116.0±5.2%), and WAT-SNA (145.7%±34.9%), but these responses were significantly less compared with those induced in the control siRNA-treated rats (Figure 3C, Figure 4C and Figure 5C).

### Sympathetic responses to ICV injection of MT-II in AMPKα2 siRNA-treated rats

**Figure 6:**
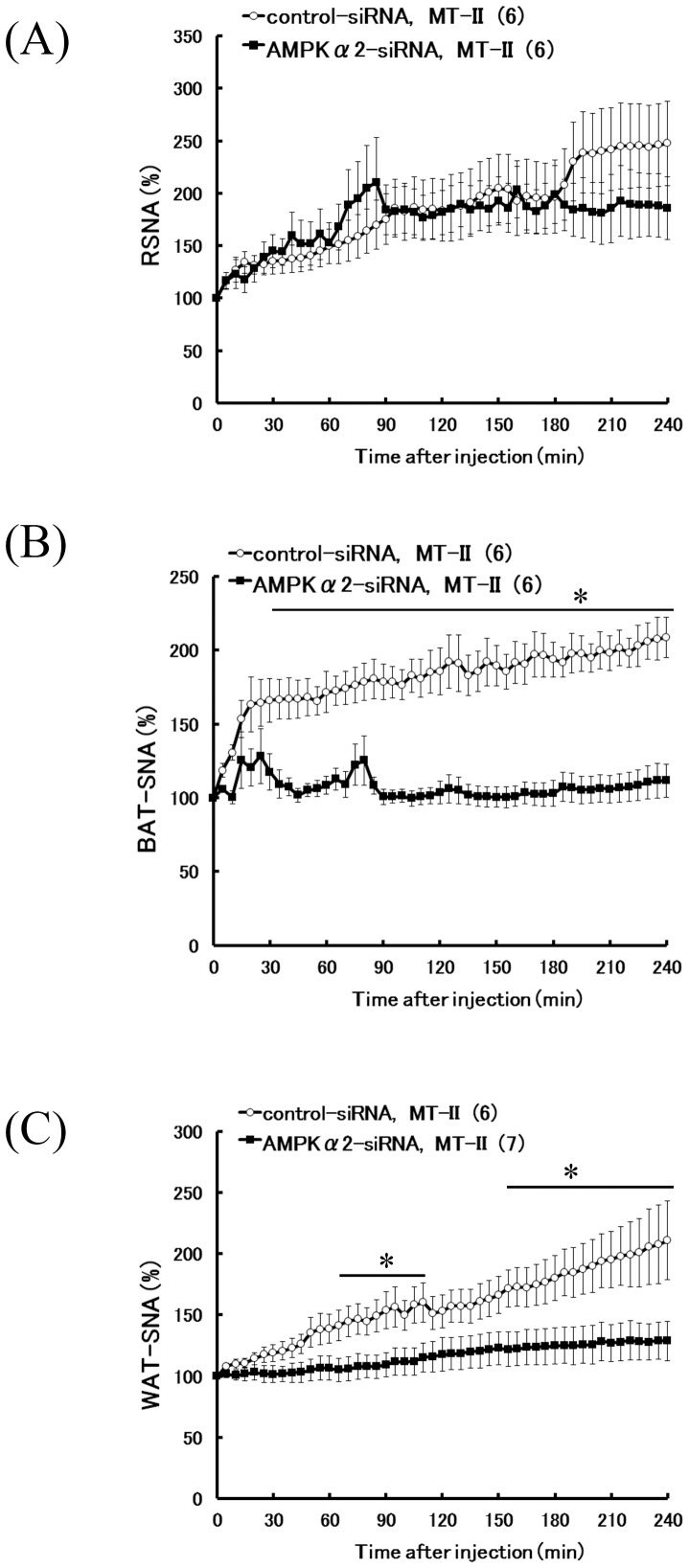
Effects of hypothalamic AMPKα2 siRNA knockdown on regional SNA following ICV injection of MT-II. Time-course data of RSNA (A), BAT-SNA (B) and WAT-SNA (C) responses to MT-II in both group (control-siRNA and AMPKα2-siRNA) were presented as mean ± SEM. Number of animals used is shown in parentheses. *P<0.05 vs. control-siRNA group.

Given that leptin increased RSNA through the activation of hypothalamic melanocortin receptors^16^, we examined the effects of MT-II, a melanocortin-3 and -4 receptor (MC3/4-R) agonist, on RSNA, BAT-SNA, and WAT-SNA in AMPKα2 siRNA-treated rats to determine if the involvement of hypothalamic AMPKα2 in autonomic control is specific to leptin signaling. MT-II administration increased RSNA similarly in both control and AMPKα2 siRNA-treated rats (Figure 6A). In contrast, MT-II injection caused greater BAT and WAT sympatho-excitations in control siRNA-treated rats as compared to AMPKα2 siRNA-treated rats. Significant differences were observed in BAT-SNA at 30–240 min after injection and in WAT-SNA at 65–110 min and 155–240 min after injection (Figure 6B and 6C).

## Discussion

Previous studies indicated that hypothalamic AMPK plays a critical role in central leptin signaling in controlling appetite and body weight [6], [7], [8]. Recently, we showed that ICV injection of Compound C, an AMPK inhibitor, dose-dependently increased the activities of the sympathetic nerves that innervated the adrenal glands and kidneys in urethane-anesthetized rats, suggesting that central nervous system AMPK contributes to sympathetic control [13]. Central nervous system AMPK includes both AMPKα1 and AMPKα2, and leptin selectively suppresses both AMPKα2 activity and the phosphorylation status of the AMPK α-sbunit [7], [19]. In the present study, we also demonstrated that knockdown of hypothalamic AMPKα2 in rats results in resistance to the enhancing actions of leptin on the sympathetic nerve outflow, cardiovascular functions and plasma FFA levels. These results support the idea that hypothalamic AMPKα2 is an important modulator of the actions of leptin on homeostasis regulation through the sympathetic nervous system. We observed an approximately 25% decrease in the hypothalamic AMPKα2 level in response to AMPKα2 knockdown, resulting in elimination of sympathetic and feeding responses to leptin; the role of the remaining approximately 75% of AMPKα2 has not been determined in the present study. Hypothalamic AMPK activity is affected by various hormones, including insulin, ghrelin and adiponectin, resulting in regulation of feeding behavior [6]. Moreover, leptin regulates not only sympathetic nerve activity but also parasympathetic nerve activity [20]. Accordingly, it is possible that the remaining approximately 75% AMPKα2 may be involved in the effects of various feeding-related hormones or in the regulation of parasympathetic nerve activity. However, further research will be needed to verify this hypothesis.

Previous studies using mouse models with altered AMPKα2 activity due to adenovirus infection or AMPKα2-deficiency implicated hypothalamic AMPKα2 in leptin-induced reduction in feeding and body weight [7], [8]. In the current study, we used an *in vivo* siRNA silencing method to reduce the expression of a targeted factor with site-specificity in the animal brain. We generated hypothalamic AMPKα2 knockdown rats and examined their feeding responses after leptin injection. Consistent with our previous idea, our present study showed that the effects of leptin on food intake and body weight were inhibited in AMPKα2 siRNA-treated rats. In AMPKα2 siRNA-treated rats, the reduction in body weight 3 days after siRNA transfection was greater than that in control siRNA rats. It is unclear why AMPKα2 knockdown by siRNA caused a body weight reduction. However, if as previously shown, the normal effect of leptin is to reduce food intake and body weight via a decrease in hypothalamic AMPKα2 activity [7], it is to be expected that AMPKα2 knockdown will also result in reduced food intake and body weight. Moreover, Claret et al. [8] found that mice lacking AMPKα2 in agouti-related protein (AgRP)-expressing neurons developed a lean phenotype. Thus, AMPKα2 knockdown by siRNA may have affected hypothalamic AMPKα2 levels in AgRP neurons, which could have resulted in the reduction in the body weight of AMPKα2 siRNA-treated rats.

Interestingly, despite the reduction in body weight of AMPKα2 siRNA-treated rats, this model exhibited elevated basal BP and HR status and attenuated BP and HR responses to leptin. Recently, it was shown that AMPKα2-deficient mice exhibited elevation in BP compared with wild type mice [11]. Moreover, IP injection of AICAR, an AMPK activator, significantly reduced the mean BP and HR of spontaneously hypertensive rats in a dose-dependent manner [21]. This evidence suggested that elevated BP and HR induced by the knockdown of hypothalamic AMPKα2 were consistent with previous observations and that hypothalamic AMPKα2 may act as a cardiovascular regulator in the brain. On the other hand, in animals with high BP, blood leptin level was elevated, resulting in leptin resistance and a reduction of leptin sensitivity [22]. Moreover, leptin injection did not stimulate BP in these animals [22]. In the present study, the lack of change in blood leptin levels in AMPKα2 siRNA-treated rats suggested that high basal levels of cardiovascular functions, but not low leptin sensitivity, may contribute to the attenuated responses of BP and HR to leptin. In support of this notion, elevated BP and HR in animals with disruptions in intracellular molecules involved in leptin signaling, such as SHP2, may be associated with the attenuated cardiovascular responses to leptin [23]. On the other hand, in the present study, it is unclear why basal levels of BP and HR increased in the AMPKα2 siRNA-treated rats. If as described previously, leptin decreases hypothalamic AMPKα2 activity [7] and the normal effects of leptin is to elevate BP and HR, it is to be expected that AMPKα2 knockdown will also result in elevated BP and HR. Moreover, we speculate that communication between the hypothalamus and the medulla may be affected by the knockdown of hypothalamic AMPKα2. In the medulla oblongata, the rostral ventrolateral medulla, which plays an important role in regulating BP and HR, has neural connections with the hypothalamus [24]. Thus, it is possible that changes in neurons of the rostral ventrolateral medulla in response to hypothalamic knockdown of the AMPKα2 are involved in abnormal cardiovascular functions such as hypertension or tachycardia.

Our observation that leptin-induced increases in sympathetic nerve discharges to kidney, BAT and WAT were eliminated in AMPKα2 siRNA-treated rats is consistent with the involvement of this pathway in the cardiovascular and weight-reducing effect of leptin. In animal models having sympatho-excitation status and hyperleptinemia, leptin-induced increase in sympathetic nerve outflow is attenuated [4], [22]. However, our present study showed no significant elevation in basal levels of plasma catecholamine and leptin in AMPKα2 siRNA-treated rats, implicating factors other than saturation of sympathetic response to leptin in the attenuated responses of sympathetic nerve activity to leptin in these rats. With regard to a possible role of an intracellular pathway in regulating RSNA by leptin, a previous study indicated that PI3-kinase plays a crucial role in leptin-evoked activation of RSNA [3]. Although the relationship between intracellular PI3-kinase and AMPK in sympathetic and cardiovascular regulation by leptin remains unclear, recent *in vitro* studies confirmed that PI3-kinase and AMPK are involved in the regulation of leptin-related NPY secretion or neuronal polarization [25], [26]. On the other hand, sympatho-excitation of BAT promotes weight loss by increasing energy expenditure [2], and increased WAT-SNA stimulates lipolysis and raises plasma levels of FFA, which are then transported to BAT for burning. In the present study, leptin injection resulted in increased plasma FFA levels in control siRNA-treated rats but did not result in an increase in FFA levels in AMPKα2 siRNA-treated rats. These results suggest that hypothalamic AMPK may play a crucial role in leptin-induced increases in energy metabolism that are mediated by sympathetic nerve activation in BAT and WAT.

A relationship between the melanocortin system and AMPK in feeding regulation has been suggested previously, mainly based on the finding that ICV injection of MT-II suppresses AMPKα2 activity in the hypothalamus [7]. In addition, ICV injection of MT-II activated RSNA and BAT-SNA in anesthetized rats and increased BAT thermogenesis [17], [27], [28], glucose uptake in BAT [29] and arterial pressure [30]. Our current study shows that silencing hypothalamic AMPKα2 eliminates BAT-SNA and WAT-SNA responses to MTII but not RSNA responses. Thus, our results suggest a role for hypothalamic AMPKα2 in thermogenesis- and lipolysis-related sympatho-regulation by the melanocortin system. Although we did not determine the detailed mechanism of RSNA control by the melanocortin system in the present study, pathways including PI3-kinase, ERK1/2, PKA, PKC, or PLC are known to be involved in neural signaling through the melanocrtin-4 receptor [31]. A previous study showed that LY294002, a PI3-kinase inhibitor, did not affect RSNA activation after ICV MT-II [3]. Thus, it is possible that a complex intracellular pathway, other than the PI3-kinase pathway, in hypothalamic neurons may be involved in RSNA control by the melanocortin system.

Leptin reduces food intake and body weight by suppressing AMPKα2 activity in the hypothalamic arcuate nucleus (ARC) and the paraventricular nucleus (PVN) [6], [7]. The underlying mechanism involves the neural actions of α-MSH derived from activated POMC neurons in ARC on MC4-R in PVN [6], [7]. In support of this notion, our results showed that appetite and body weight suppression by ICV leptin are abolished in AMPKα2 siRNA-treated rats. However, we did not determine whether AMPK suppression in ARC or PVN played an important role in sympatho-excitation by leptin. Recently, microinjection studies demonstrated that the direct action of leptin on the ARC in the hypothalamus is important for stimulating RSNA and BAT-SNA [5], and mice with adenovirus-mediated ARC leptin receptor ablation have attenuated RSNA and BAT-SNA responses to leptin [32]. These results suggest that central leptin may first target ARC in the hypothalamus and generate neurotransmission involving AMPK or other neuropeptides to stimulate the sympathetic nerve tone. Although additional studies will be needed to determine whether AMPK activity in ARC has a crucial role in leptin-induced sympatho-excitation, our present study suggests that the hypothalamic AMPK pathway has a crucial role in the cardiovascular, thermogenic and lipocatabolic actions of leptin in mediating the stimulation of sympathetic innervations of kidney, BAT and WAT.

In conclusion, in the present study, we have shown that hypothalamic AMPK is implicated in cardiovascular, thermogenic, and lipid-metabolic actions of leptin through sympathetic activation. Our results provide novel insight into the molecular mechanisms involved in the hypothalamic action of leptin in the control of the sympathetic nervous system. Previous studies have shown that major intracellular signaling pathways, such as those involving MAP-kinase or PI3-kinase, are mediated by sympathetic responses to leptin [2], [3]. MAP-kinase and PI3-kinase in hypothalamic neurons may also affect hypothalamic AMPK activity and selectively regulate sympathetic nerve outflows to various tissues, including the kidney, adipose tissue and other abdominal organs. Thus, in order to selectively evaluate leptin actions on the cardiovascular system, thermoregulation, energy metabolism and feeding behavior, it will be necessary to examine intracellular signaling in terms of major upstream pathways and the activities of downstream factors.
